# Intravenous iron for heart failure with evidence of iron deficiency: a meta-analysis of randomised trials

**DOI:** 10.1007/s00392-021-01837-8

**Published:** 2021-03-23

**Authors:** Fraser J. Graham, Pierpaolo Pellicori, Ian Ford, Mark C. Petrie, Paul R. Kalra, John G. F. Cleland

**Affiliations:** 1 Robertson Centre for Biostatistics, University of Glasgow, Boyd Orr Building, University Avenue, Glasgow, UK; 2 British Heart Foundation Cardiovascular Research Centre, University of Glasgow, Glasgow, UK; 3 Department of Cardiology, Portsmouth Hospitals University NHS Trust, Portsmouth, UK

**Keywords:** Iron deficiency, Heart failure, Intravenous iron, Meta-analysis

## Abstract

**Background:**

The recent AFFIRM-AHF trial assessing the effect of intravenous (IV) iron on outcomes in patients hospitalised with worsening heart failure who had iron deficiency (ID) narrowly missed its primary efficacy endpoint of recurrent hospitalisations for heart failure (HHF) or cardiovascular (CV) death. We conducted a meta-analysis to determine whether these results were consistent with previous trials.

**Methods:**

We searched for randomised trials of patients with heart failure investigating the effect of IV iron vs placebo/control groups that reported HHF and CV mortality from 1st January 2000 to 5th December 2020. Seven trials were identified and included in this analysis. A fixed effect model was applied to assess the effects of IV iron on the composite of first HHF or CV mortality and individual components of these.

**Results:**

Altogether, 2,166 patients were included (*n* = 1168 assigned to IV iron; *n* = 998 assigned to control). IV iron reduced the composite of HHF or CV mortality substantially [OR 0.73; (95% confidence interval 0.59–0.90); *p* = 0.003]. Outcomes were consistent for the pooled trials prior to AFFIRM-AHF. Whereas first HHF were reduced substantially [OR 0.67; (0.54–0.85); *p* = 0.0007], the effect on CV mortality was uncertain but appeared smaller [OR 0.89; (0.66–1.21); *p* = 0.47].

**Conclusion:**

Administration of IV iron to patients with heart failure and ID reduces the risk of the composite outcome of first heart failure hospitalisation or cardiovascular mortality, but this outcome may be driven predominantly by an effect on HHF. At least three more substantial trials of intravenous iron are underway.

**Graphic abstract:**

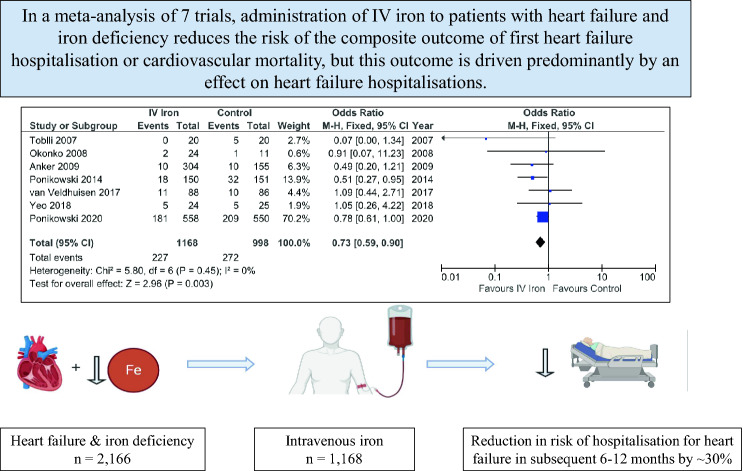

**Supplementary Information:**

The online version contains supplementary material available at 10.1007/s00392-021-01837-8.

## Introduction

Patients with heart failure often have evidence of iron deficiency (ID), with or without anaemia, which is associated with more severe symptoms, lower exercise capacity and higher rates of hospitalisations for heart failure (HHF) and mortality [1, 2]. In an individual patient meta-analysis of four trials including 839 patients with heart failure with reduced ejection fraction (HFrEF) and serum markers of ID, Anker and colleagues suggested that administration of intravenous iron (IV) reduced the risk of first and recurrent HHF when compared to placebo [3]. Recently, the AFFIRM-AHF trial narrowly missed its primary efficacy endpoint of recurrent HHF or cardiovascular (CV) death [4]. Therefore, we produced an updated the meta-analysis to investigate whether the effects of IV iron were consistent amongst the randomised trials reported so far and whether sufficient evidence had accumulated to indicate a conclusive effect on HHF and CV mortality.

## Methods

We searched for English language trials from 1st January 2000 to 5th December 2020 in PubMed using pre-specified search terms (see Supplements), and from additional sources including a recent systematic review [5]. Only published randomised trials investigating the effects of IV iron compared to a control group that did not receive IV iron in patients with heart failure, regardless of participants’ left ventricular ejection fraction, the formulation of IV iron, concomitant therapy, or definition of ID, that reported either HHF or CV mortality were included in the main report. If mortality was not explicitly reported but HHF was, it was assumed that no deaths had occurred. An additional analysis was done including two unpublished trials, with data derived from the meta-analysis reported by Anker et al. [3].

Data were extracted by two independent reviewers (FG and PP). Deaths not clearly declared as CV or non-CV were adjudicated independently by two authors, both of whom are experienced in clinical end-point adjudication. Adjudication was based on the clinical information provided by authors in the text. Disparities were resolved by discussion or by checking with a third author (JGFC). Outcomes assessed were the composite of HHF or CV mortality as first events, and HHF as a first event and CV mortality separately. Data analysed were the numbers of first events and numbers of participants in each treatment arm for each trial. Odds ratios and 95% confidence intervals for the effect of treatment with IV iron relative to control were calculated for each trial. The data were analysed using both fixed effects (primary analysis) and random effects models. Forest plots with odds ratios and corresponding (95% confidence intervals) were produced and reported. A level of significance of 5% was considered statistically significant.

To assess the impact of results from the largest trial to date, additional analysis comparing odds ratios for studies excluding AFFIRM-AHF to the AFFIRM-AHF trial alone were carried out.

All analyses were conducted with Review Manager (RevMan) Version 5.4 (The Cochrane Collaboration, 2020).

## Results

**Fig. 1:**
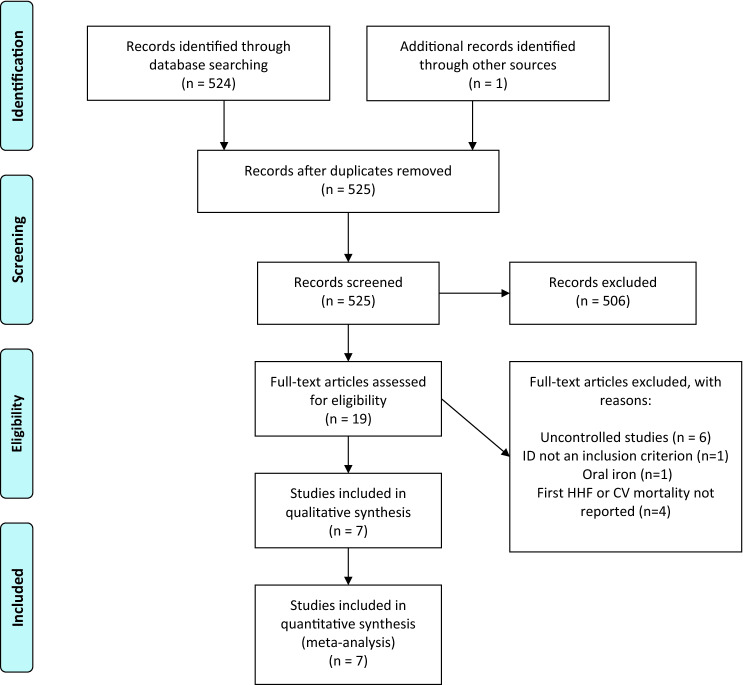
PRISMA diagram detailing the number of records identified, screened, included, and excluded, with a summary of the reasons for exclusion. Modified from Moher D, Liberati A, Tetzlaff J, Altman DG, The PRISMA Group (2009). Preferred Reporting Items for Systematic Reviews and Meta-Analyses: the PRISMA statement. PLoS Med 6(7): e1000097. 10.1371/journal.pmed1000097. *ID* iron deficiency, *HHF* hospitalisation for heart failure, *CV* cardiovascular

We identified 19 reports assessing the effect of iron therapy in patients with heart failure (Fig. 1). After excluding 12 reports [6–17], mainly because they were not randomised-controlled trials or did not report relevant outcomes (Supplementary Table S1), seven trials (Table 1) that enrolled 2,166 patients (*n* = 1168 assigned to IV iron; *n* = 998 assigned to the control/placebo) were included in the primary analysis [4, 18–23]. The most common definition of ID was a ferritin < 100 µg/L and/or, if ferritin was 100–300 µg/L, a TSAT of < 20%. Most trials excluded patients with a very low haemoglobin (less than 8–10 g/dL) or with values greater than 15 g/dL [4, 19]. Only two trials followed patients for > 6 months [4, 19]. Five trials used ferric carboxymaltose and two used iron sucrose [21, 22].

**Table 1:** Characteristics of included trials

		Toblli et al	FERRIC-HF	FAIR-HF	CONFIRM-HF	EFFECT-HF	PRACTICE- ASIA-HF	AFFIRM-AHF
Year of publication		2007	2008	2009	2014	2017	2018	2020
Country		Argentina	UK and Poland	Europe and Argentina	Europe	Europe and Australia	Singapore	15 countries (International)
Number of patients (IV iron: control)		40 (1:1)	35 (2:1)	459 (2:1)	301 (1:1)	174 (1:1)	49 (1:1)	1108 (1:1)
Double-blind		Yes	No	Yes	Yes	No	No	Yes
Definition of ID		*F* < 100 and/or *T* ≤ 20%	*F* < 100 or *T* < 20% + F100–300	*F* < 100 or *T* < 20% + F100–299	*F* < 100 or *T* < 20% + F100–300	*F* < 100 or T < 20% + F100–300	*T* < 20% and *F* < 300	*F* < 100 or *T* < 20% + F 100–299
Main inclusion criteria (Hb: g/dL)		• LVEF ≤ 35% • NYHA II-IV • Anaemia	• LVEF ≤ 45% • NYHA II-III • Hb ≤ 14.5	• LVEF ≤ 45% • NYHA II-III • Hb 9.5–13.5	• LVEF ≤ 45% • NYHA II/III • Hb < 15	• LVEF ≤ 45% • NYHA II or III • Hb < 15	• HF Hosp • Hb < 14	• LVEF < 50% • HF Hosp • NT-proBNP↑ • Hb 8–15
Age (years)		75	63	68	70	64	63	71
Women (%)		–	29	54	47	25	22	45
Ischaemic aetiology (%)		63	74	80	83	–	–	47
LVEF (%)		31 ± 4	30 ± 7	32 ± 6	37 ± 8	33 ± 9	39 ± 18	33 (10)
NT-proBNP (pg/ml)		256 ± 125	–	–	2511 ± 5006	1576*	–	4743 (2781–8128)*
eGFR (ml/min/1.73m^2^)		–	–	64	66	52	–	–
Haemoglobin (g/dL)		10.3 ± 0.6	12.6 ± 1.2	11.9 ± 1.3	12.3 ± 1.4	12.9 ± 1.3	11.6 ± 1.9	12.3 ± 1.6
Ferritin (µg/L)		73 ± 30	62 ± 37	53 ± 55	57 ± 48	48*	91 ± 80	84 ± 62
TSAT (%)		20 ± 1	20 ± 8	18 ± 13	20 ± 18	17*	16 ± 10	15 ± 8
Form of iron therapy (mean dose)		Iron sucrose; 1000 mg	Iron sucrose; 1433 mg	FCM; n/a	FCM; 1500 mg	FCM; 1204 mg	FCM; 1000 mg	FCM; 1352 mg
Follow-up		24 weeks	18 weeks	24 weeks	52 weeks	24 weeks	12 weeks	52 weeks
Outcomes reported	HHF	+	+	+	+	+	+	+
	CVM	−	+	+	+ ^a^	+	−	+

Data shown are for the active group only, but this is also representative of the control group. Data presented as mean ± SD or count and (%) unless otherwise stated

If data not available/reported, cell filled (–). *Median and (Q1–Q3) reported

^a^Not specifically reported but derived from reported outcomes in the paper and from the individual-patient-data meta-analysis by Anker et al.—see reference [3]

*ID* iron deficiency, *F* ferritin (µg/L), *T* transferrin saturation (%), *LVEF* left ventricular ejection fraction, *NYHA* New York Heart Association, *Hb* haemoglobin, *NT-proBNP* N-terminal pro-brain natriuretic peptide, *IV* intravenous, *eGFR* estimated glomerular filtration rate, *FCM* ferric carboxymaltose, *HHF* hospitalisation for heart failure, *CVM* cardiovascular mortality

**Fig. 2:**
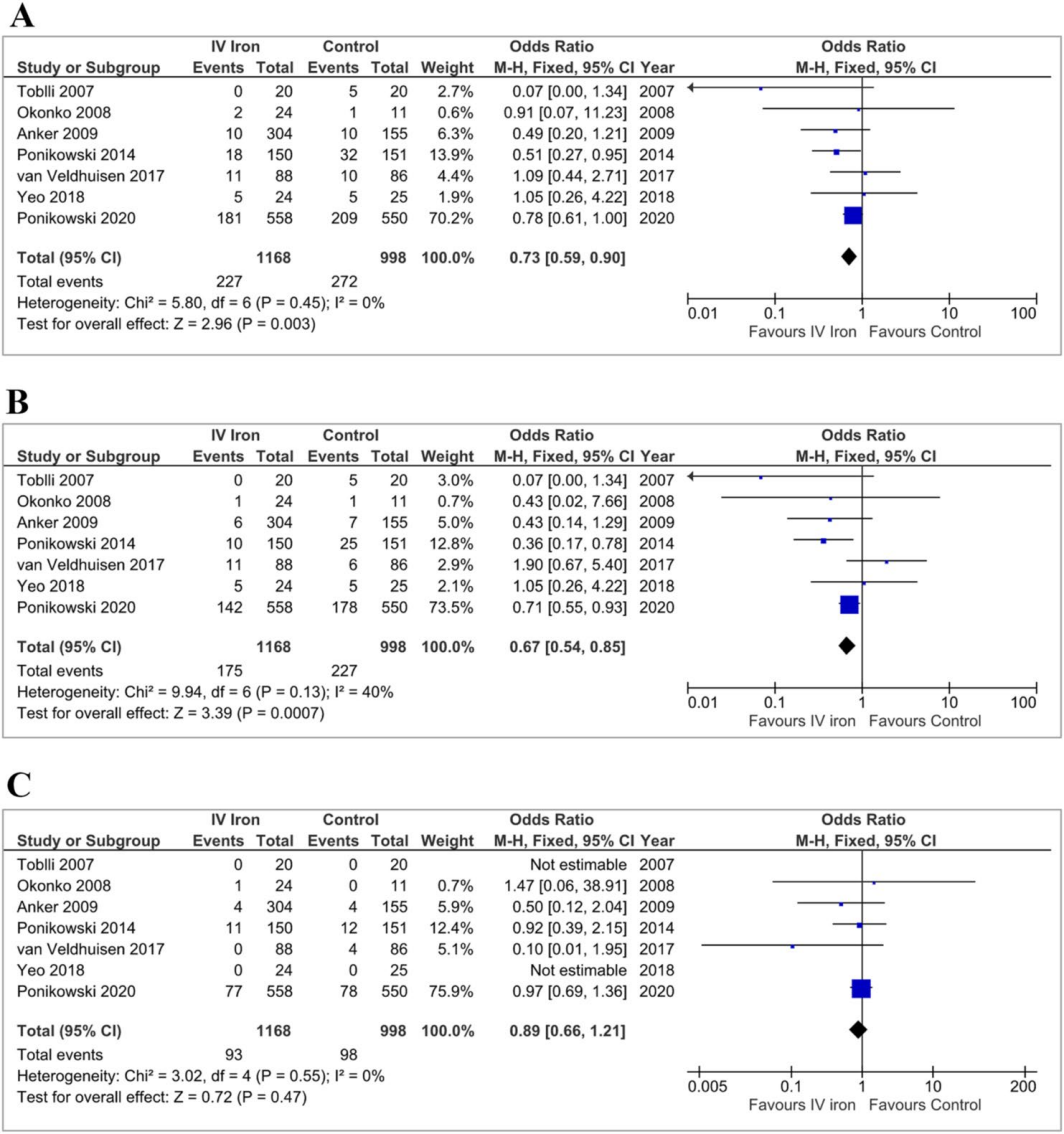
Fixed-effects meta-analysis model of all included trials detailing the pooled effect of intravenous iron on the composite of first hospitalisations for heart failure or cardiovascular mortality (**a**), and first hospitalisation for heart failure (**b**) and cardiovascular mortality (**c**) alone. *IV* intravenous, *CI* confidence interval

In the primary analysis, IV iron reduced the composite outcome of HHF or CV death: OR 0.73 [0.59–0.90]; *p* = 0.003 (Fig. 2a). HHF occurred in 175 (15%) patients administered IV iron and 227 (23%) assigned to control: OR 0.67 [0.54–0.85]; *p* = 0.0007 (Fig. 2b). CV deaths occurred in 93 (8%) patients administered IV iron and in 98 (10%) assigned to control: OR 0.89 [0.66–1.21]; *p* = 0.47 (Fig. 2c). Adding data from the two unpublished trials to the main analysis did not substantially alter these results (Supplementary Figure S1).

**Fig. 3:**
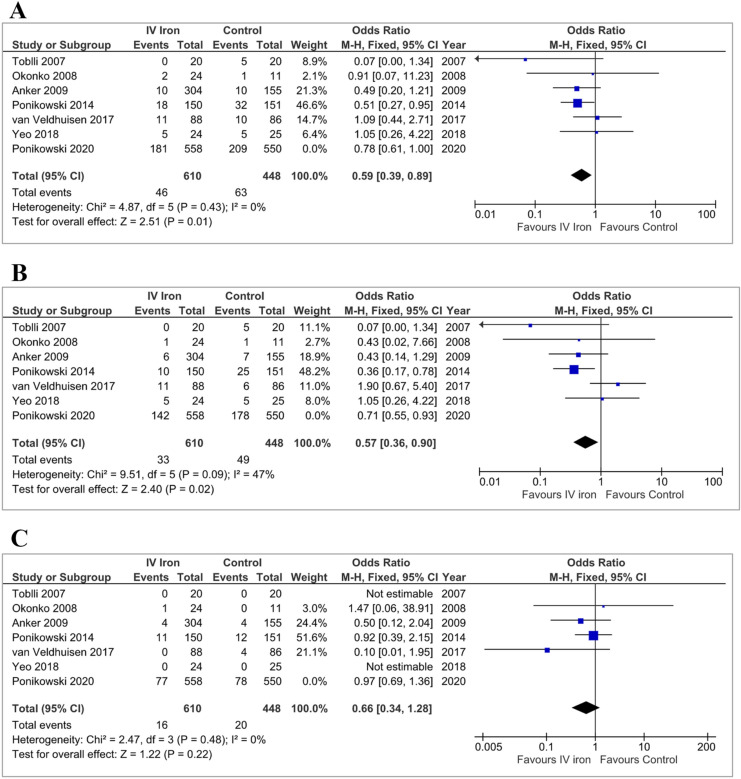
Fixed-effects meta-analysis model of all trials, excluding AFFIRM-AHF, detailing the pooled effect of intravenous iron on the composite of first hospitalisations for heart failure or cardiovascular mortality (**a**), and first hospitalisation for heart failure (**b**) and cardiovascular mortality (**c**) alone. Although not included in the pooled analysis, odds ratios and 95% confidence intervals are presented for AFFIRM-AHF for comparison. *IV* intravenous, *CI* confidence interval

When AFFIRM-AHF was excluded from the model, the point estimates for the effect of IV iron were OR 0.59 [0.39–0.89]; *p* = 0.01 for the composite outcome, OR 0.57 [0.36–0.90]; *p* = 0.02 for HHF and OR 0.66 [0.34–1.28]; *p* = 0.22 for CV mortality (Table 2 and Fig. 3). The odds ratios for all outcomes were not significantly different for the pooled data excluding AFFIRM-AHF compared to AFFIRM-AHF alone (Table 3).

**Table 2:** Summary of results from meta-analysis models, with and without AFFIRM-AHF, and AFFIRM-AHF alone, assessing the effect of IV iron on outcomes

Outcome	IV iron		Controls		Fixed effect		Random effect	
	Events	Patients	Events	Patients	OR (95% CI)	*p*	OR (95% CI)	*p*
AFFIRM-AHF excluded								
CVM or HHF	46	610	63	448	0.59 (0.39, 0.89)	0.01	0.62 (0.41, 0.93)	0.02
HHF	33	610	49	448	0.57 (0.36, 0.90)	0.02	0.60 (0.28, 1.28)	0.19
CVM	16	610	20	448	0.66 (0.34, 1.28)	0.22	0.72 (0.36, 1.43)	0.35
AFFIRM-AHF								
CVM or HHF	181	558	209	550	0.78 (0.61, 1.00)	–	0.78 (0.61, 1.00)	–
HHF	142	558	178	550	0.71 (0.55, 0.93)	–	0.71 (0.55, 0.93)	–
CVM	77	558	78	550	0.97 (0.69, 1.36)	–	0.97 (0.69, 1.36)	–
All Trials								
CVM or HHF	227	1168	272	998	0.73 (0.59, 0.90)	0.003	0.74 (0.60–0.91)	0.005
HHF	175	1168	227	998	0.67 (0.54, 0.85)	0.0007	0.64 (0.40, 1.04)	0.07
CVM	93	1168	98	998	0.89 (0.66, 1.21)	0.47	0.91 (0.67, 1.24)	0.56

*IV* intravenous, *OR* odds ratio, *CI* confidence interval, *CVM* cardiovascular mortality, *HHF* hospitalisation for heart failure

**Table 3:** Comparison of fixed-effects odds ratios (ORs) between pooled trials prior to AFFIRM-AHF and AFFIRM-AHF

	Odds ratio (95% confidence interval)	*P* for comparison	OR (95% confidence interval) of comparison
Composite endpoint			
All trials except AFFIRM-AHF	0.59 (0.39, 0.89)	0.26	0.76 (0.47, 1.22)
AFFIRM-AHF	0.78 (0.61, 1.00)		
Hospitalisation for heart failure			
All trials except AFFIRM-AHF	0.57 (0.36, 0.90)	0.41	0.80 (0.47, 1.36)
AFFIRM-AHF	0.71 (0.55, 0.93)		
Cardiovascular mortality			
All trials except AFFIRM-AHF	0.66 (0.34, 1.28)	0.31	0.68 (0.32, 1.43)
AFFIRM-AHF	0.97 (0.69, 1.36)		

In random effects models, IV iron reduced the composite outcome [OR 0.74 (0.60–0.91); *p* = 0.005] but neither HHF [OR 0.64 (0.40–1.04); *p* = 0.07] nor CV mortality [OR 0.91 (0.67–1.24); *p* = 0.56] (Supplementary Figure S2). Results were similar when AFFIRM-AHF was excluded (Table 2 and Supplementary Figure S3).

## Discussion

This meta-analysis suggests that IV iron reduces the risk of the composite outcome of first HHF or CV death for patients with serum markers of ID and heart failure. This result was driven predominantly by an effect on HHF with no convincing evidence of a reduction in CV mortality. Because HHF is associated with a higher risk of CV mortality, the effect of IV iron for each outcome might be expected to be rather similar. The relatively small number of deaths, the short duration of follow-up and the play of chance might explain this possible anomaly. A longer duration of follow-up might show a greater effect on CV mortality, but the trial with the longest follow-up to date, albeit only one year, showed little effect on this outcome [4]. The AFFIRM-AHF trial suggests that the reduction in hospitalisations for heart failure is not observed until 8–12 weeks after administration of IV iron, consistent with its benefits being mediated through the synthesis of new red blood cells, myoglobin and other metalloproteins. Accordingly, large effects observed in some small trials lasting 3 months or less may reflect chance effects.

The effects of IV iron appeared somewhat greater in a pooled analysis of trials excluding AFFIRM-AHF, although differences were not statistically significant. IV iron might be more effective in clinically stable populations. Differences in study design, inclusion criteria, iron dosing and length of follow-up may affect the outcome. The small size of some trials confounded statistical assessment of heterogeneity. Instead, we produced both fixed and random effects models which, for the composite outcome, yielded similar results, although less secure for effects on HHF in the random effects model.

Whether the definition of ID used in these trials is optimal is uncertain. Using a TSAT < 20% alone might be a better guide to ID than one based on ferritin [24–26]. This is important because giving IV iron to patients who are not iron-deplete is unlikely to be beneficial. Fortunately, ID appears common in patients with heart failure and therefore an effect might be detected even if the diagnostic accuracy of the test for ID is poor. Perhaps most patients with heart failure have ID and the key question is how severe it is, rather than whether it is present; ID should not be a binary, all-or-nothing classification.

We did not conduct subgroup analyses, which are best left to an individual-patient-data (IPD) meta-analysis that can adjust for confounding variables. In an IPD meta-analysis [3], lower TSAT but not lower serum ferritin predicted greater benefit from IV iron. In AFFIRM-AHF it appeared that lower serum ferritin or a lower TSAT were associated with greater benefit from IV iron, but > 80% of participants had a TSAT < 20%. Further analyses are required. Haemoglobin concentration has not predicted benefit but, because women have lower concentrations than men, such analyses may be confounded by participants’ sex. The AFFIRM-AHF trial enrolled patients with new-onset heart failure, which is unusual for trials of heart failure; these patients may have had somewhat less benefit from IV iron possibly because they were less likely to have true ID or because the determinants of outcome in such patients are different. In AFFIRM-AHF patients with ischaemic cardiomyopathy appeared to have greater benefit; the reasons for this are unclear. The reduction in events with IV iron, compared to control, might have been underestimated because treatments for heart failure might have been more likely to be intensified in the control group who did not receive the symptomatic benefits of iron therapy. This possibility should be explored in the future analysis of substantial long-term trials.

Results from three other large ongoing trials should clarify the effects of IV iron on morbidity and mortality in patients with HFrEF and ID and provide further insights into the possible predictors of response [27]. Trials in heart failure with preserved ejection fraction are also underway but limited data currently exist [23].

### Limitations

We did not investigate the effect of IV iron on all-cause mortality as this is not yet reported for AFFIRM-AHF. The composite outcome reported for CONFIRM-HF [19] was HHF and all-cause mortality, which included one non-CV death amongst patients assigned to iron and two to placebo. This would not materially alter our overall results. An analysis of recurrent HHF rather than just the first event would make the result more robust but requires access to IPD. An IPD meta-analysis has many advantages when exploring the interaction amongst variables [28–30]. In particular, an IPD would have allowed analysis of the potential interaction between sex and the effects of IV iron. However, aggregate data have the advantage that it includes all the published data rather than the proportion where IPD is available to the authors. Each type of meta-analysis has advantages, and they are complimentary. All meta-analyses should be interpreted cautiously, particularly for analyses involving a number of small studies where there will be little power to detect heterogeneity. Fixed effects meta-analysis provides an estimate of an average treatment effect in the studies conducted but uncertainty about heterogeneity may make it difficult to extrapolate that effect to a particular clinical context. Random effects analyses assume that studies have underlying treatment effects arising from a random distribution and provide estimates of the average of, and variation in, the treatment effect in that distribution. However, if the variation is systematic and not random then the random effects analysis may not be helpful in extrapolating a treatment effect to a new situation. In the context of this analysis, length of follow-up, clinical status of patients at recruitment and IV iron dosing strategy are systematically different amongst the studies. Whether these factors systematically impact the treatment effect is difficult to determine with the data available.

## Conclusion

In a meta-analysis of seven trials, administration of IV iron to patients with heart failure and ID reduced the risk of the composite outcome of heart failure hospitalisation or cardiovascular mortality in the following 12 months. To date, this outcome is driven predominantly by an effect on HHF. Longer term effects of repeated administration of IV iron are unknown. More evidence is desirable.

## Supplementary Information

Below is the link to the electronic supplementary material.

Supplementary file1 (DOCX 21 KB)

Supplementary file2 (PPTX 465 KB)

Supplementary file3 (PPTX 450 KB)

Supplementary file4 (PPTX 456 KB)

## Data Availability

Not applicable.
