# Supplementary material for: Intravenous iron for heart failure with evidence of iron deficiency: a meta-analysis of randomised trials
Source: Clin Res Cardiol. 2021 Mar 23;110(8):1299–307. doi: 10.1007/s00392-021-01837-8 (PMC8318946; doi:10.1007/s00392-021-01837-8)
Supplement: Supplementary file 1 — Supplementary file1 (DOCX 21 KB) [file 392_2021_1837_MOESM1_ESM.docx]

**Search terms**

Pre-specified search terms: ((Heart failure) and (iron deficiency) OR (iron repletion) OR (intravenous iron) OR (ferric carboxymaltose) OR (iron sucrose) OR (iron supplementation) OR (iron therapy))

Filters: English Language; Clinical trial; Randomized trial

Date range: 1^st^ January 2000 – 5^th^ December 2020

| **Table S1: Excluded Trials and reasons for exclusion** | |
| --- | --- |
| Reason for exclusion | Trial |
| Uncontrolled studies  (n= 6) | Silverberg DS, Wexler D, Blum M, Tchebiner JZ, Sheps D, Keren G, Schwartz D, Baruch R, Yachnin T, Shaked M, Schwartz I, Steinbruch S, Iaina A. The effect of correction of anaemia in diabetics and non-diabetics with severe resistant congestive heart failure and chronic renal failure by subcutaneous erythropoietin and intravenous iron. Nephrol Dial Transplant. 2003;18(1):141-6. doi: 10.1093/ndt/18.1.141.  Silverberg DS, Wexler D, Blum M, Schwartz D, Keren G, Sheps D, Iaina A. Effect of correction of anemia with erythropoietin and intravenous iron in resistant heart failure in octogenarians. Isr Med Assoc J. 2003;5(5):337-9.  Bolger AP, Bartlett FR, Penston HS, O'Leary J, Pollock N, Kaprielian R,  Chapman CM. Intravenous iron alone for the treatment of anemia in patients with chronic heart failure. J Am Coll Cardiol. 2006;48(6):1225-7. doi:10.1016/j.jacc.2006.07.015. Epub 2006 Aug 28.  Usmanov RI, Zueva EB, Silverberg DS, Shaked M. Intravenous iron without erythropoietin for the treatment of iron deficiency anemia in patients with moderate to severe congestive heart failure and chronic kidney insufficiency. J Nephrol. 2008;21(2):236-42.  Reed BN, Blair EA, Thudium EM, Waters SB, Sueta CA, Jensen BC, Rodgers JE. Effects of an accelerated intravenous iron regimen in hospitalized patients with advanced heart failure and iron deficiency. Pharmacotherapy. 2015;35(1):64-71. doi: 10.1002/phar.1525. Epub 2014 Dec 29.  Mirdamadi A, Arefeh A, Garakyaraghi M, Pourmoghadas A. Beneficial effects of the treatment of iron deficiency on clinical condition, left ventricular function, and quality of life in patients with chronic heart failure. Acta Biomed. 2018;89(2):214-218. doi: 10.23750/abm.v89i2.5020. |
| Iron deficiency not an inclusion criterion  (n= 1) | Silverberg DS, Wexler D, Sheps D, Blum M, Keren G, Baruch R, Schwartz D, Yachnin T, Steinbruch S, Shapira I, Laniado S, Iaina A. The effect of correction of mild anemia in severe, resistant congestive heart failure using subcutaneous erythropoietin and intravenous iron: a randomized controlled study. J Am Coll Cardiol. 2001;37(7):1775-80. doi: 10.1016/s0735-1097(01)01248-7. |
| Oral iron  (n= 1) | Lewis GD, Malhotra R, Hernandez AF, McNulty SE, Smith A, Felker GM, Tang WHW, LaRue SJ, Redfield MM, Semigran MJ, Givertz MM, Van Buren P, Whellan D, Anstrom KJ, Shah MR, Desvigne-Nickens P, Butler J, Braunwald E; NHLBI Heart Failure Clinical Research Network. Effect of Oral Iron Repletion on Exercise Capacity in Patients With Heart Failure With Reduced Ejection Fraction and Iron Deficiency:  The IRONOUT HF Randomized Clinical Trial. JAMA. 2017;317(19):1958-1966. doi: 10.1001/jama.2017.5427. |
| First HFH and/or CV mortality not reported  (n= 4) | Beck-da-Silva L, Piardi D, Soder S, Rohde LE, Pereira-Barretto AC, de Albuquerque D, Bocchi E, Vilas-Boas F, Moura LZ, Montera MW, Rassi S, Clausell N. IRON-HF study: a randomized trial to assess the effects of iron in heart failure patients with anemia. Int J Cardiol. 2013;168(4):3439-42. doi: 10.1016/j.ijcard.2013.04.181. Epub 2013 May 13.  Toblli JE, Di Gennaro F, Rivas C. Changes in Echocardiographic Parameters in Iron Deficiency Patients with Heart Failure and Chronic Kidney Disease Treated with Intravenous Iron. Heart Lung Circ. 2015;24(7):686-95. doi: 10.1016/j.hlc.2014.12.161. Epub 2015 Jan 21.  Charles-Edwards G, Amaral N, Sleigh A, Ayis S, Catibog N, McDonagh T, Monaghan M, Amin-Youssef G, Kemp GJ, Shah AM, Okonko DO. Effect of Iron Isomaltoside on Skeletal Muscle Energetics in Patients With Chronic Heart Failure and Iron Deficiency. Circulation. 2019;139(21):2386-2398. doi: 10.1161/CIRCULATIONAHA.118.038516.  Núñez J, Miñana G, Cardells I, Palau P, Llàcer P, Fácila L, Almenar L, López-Lereu MP, Monmeneu JV, Amiguet M, González J, Serrano A, Montagud V, López-Vilella R, Valero E, García-Blas S, Bodí V, de la Espriella-Juan R, Lupón J, Navarro J, Górriz JL, Sanchis J, Chorro FJ, Comín-Colet J, Bayés-Genís A; Myocardial‐IRON Investigators. Noninvasive Imaging Estimation of Myocardial Iron Repletion Following Administration of Intravenous Iron: The Myocardial-IRON Trial. J Am Heart Assoc. 2020;9(4):e014254. doi: 10.1161/JAHA.119.014254. Epub 2020 Feb 13. |
