# Supplementary figures and images for: Intravenous iron for heart failure with evidence of iron deficiency: a meta-analysis of randomised trials
Source: Clin Res Cardiol. 2021 Mar 23;110(8):1299–307. doi: 10.1007/s00392-021-01837-8 (PMC8318946; doi:10.1007/s00392-021-01837-8)

## Slide 1
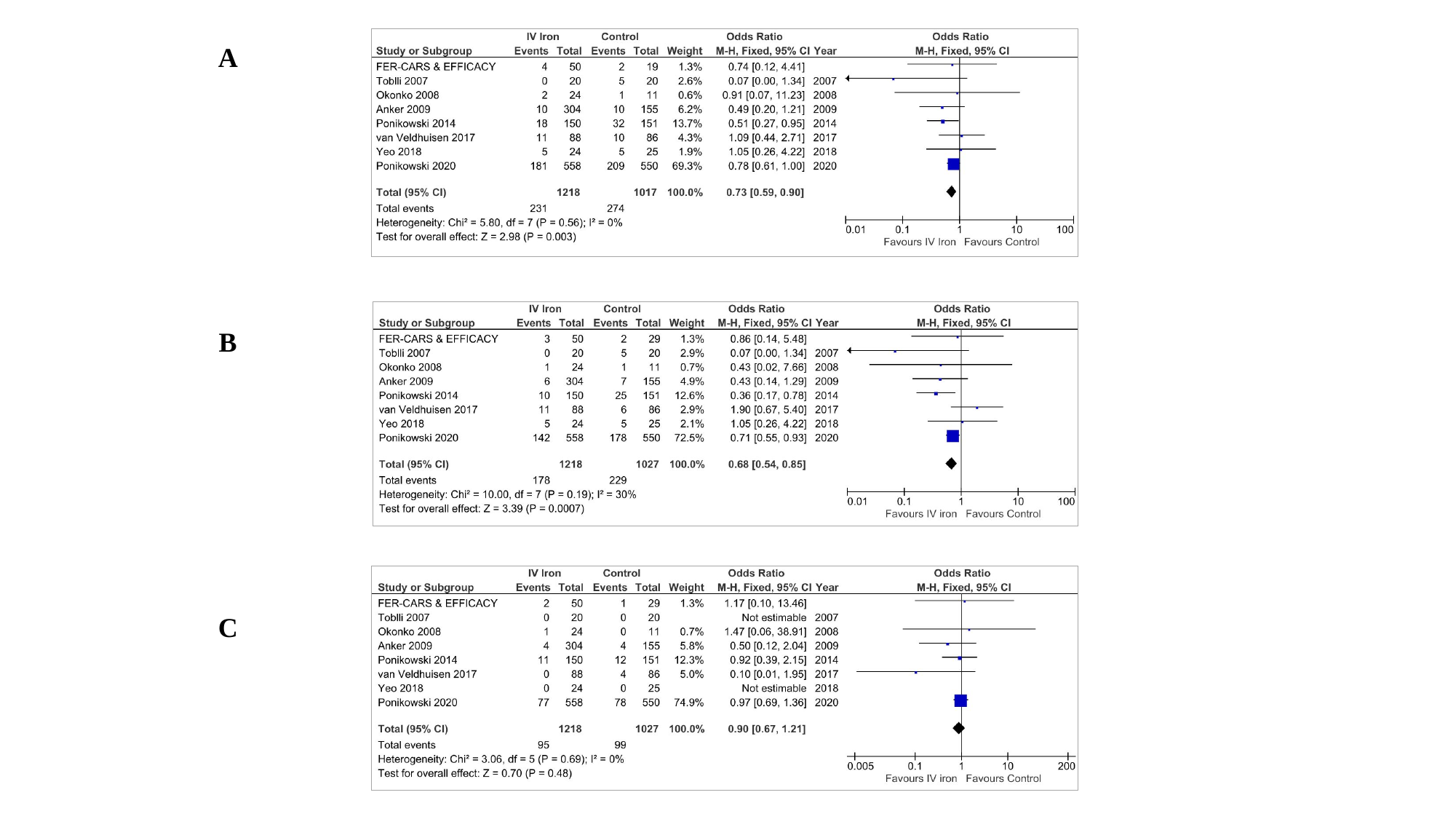

A
B
C

Supplement: Supplementary file 2 — Supplementary file2 (PPTX 465 KB) [file 392_2021_1837_MOESM2_ESM.pptx]

## Slide 1
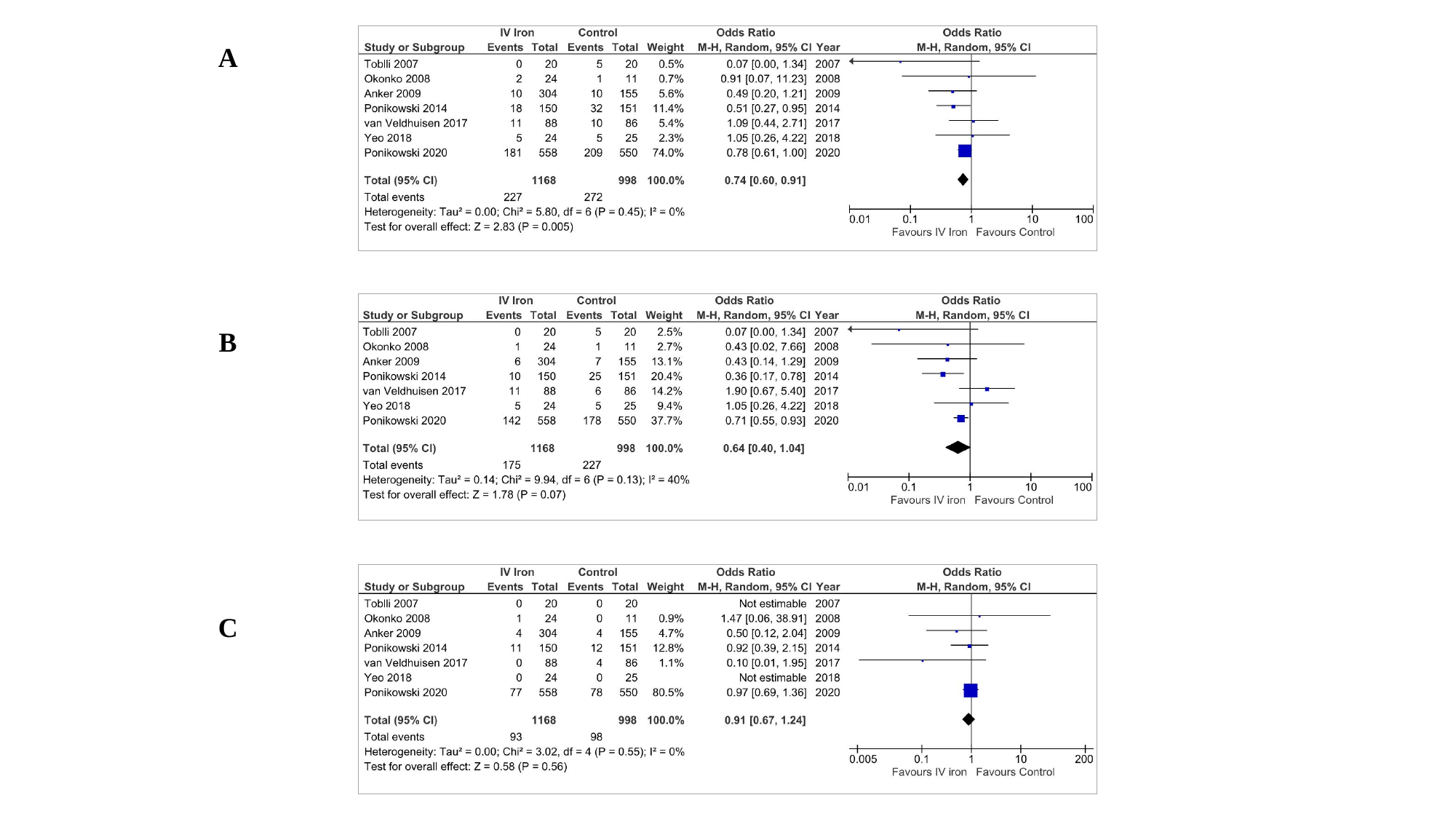

A
B
C

Supplement: Supplementary file 3 — Supplementary file3 (PPTX 450 KB) [file 392_2021_1837_MOESM3_ESM.pptx]

## Slide 1
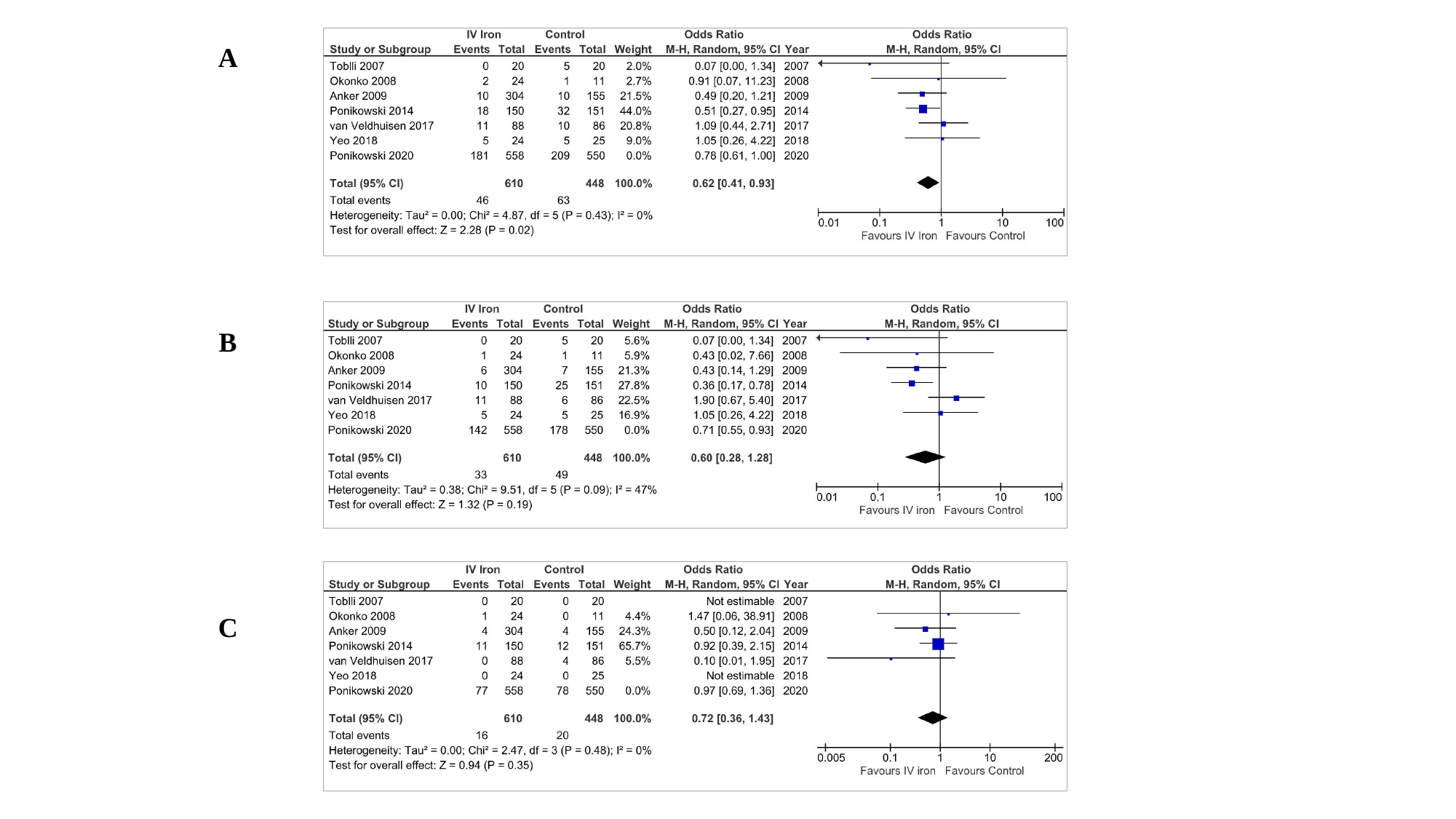

A
B
C

Supplement: Supplementary file 4 — Supplementary file4 (PPTX 456 KB) [file 392_2021_1837_MOESM4_ESM.pptx]
